# A multiplex preclinical model for adenoid cystic carcinoma of the salivary gland identifies regorafenib as a potential therapeutic drug

**DOI:** 10.1038/s41598-017-11764-2

**Published:** 2017-09-12

**Authors:** Chen Chen, Sujata Choudhury, Darawalee Wangsa, Chamille J. Lescott, Devan J. Wilkins, Praathibha Sripadhan, Xuefeng Liu, Danny Wangsa, Thomas Ried, Christopher Moskaluk, Michael J. Wick, Eric Glasgow, Richard Schlegel, Seema Agarwal

**Affiliations:** 1 Department of Pathology, Center for Cell Reprogramming, Georgetown University Medical Center, Washington, DC 20007 USA; 2 Center for Cancer Research, National Cancer Institute, Bethesda, MD 20892 USA; 3 Department of Pathology, University of Virginia, Charlottesville, VA 22903 USA; 4 START, San Antonio, TX 78229 USA; 5 Department of Oncology, Lombardi Cancer Center, Georgetown University Medical Center, Washington, DC 20007 USA

## Abstract

Adenoid cystic carcinomas (ACC) are rare salivary gland cancers with a high incidence of metastases. In order to study this tumor type, a reliable model system exhibiting the molecular features of this tumor is critical, but none exists, thereby inhibiting *in-vitro* studies and the analysis of metastatic behavior. To address this deficiency, we have coupled an efficient method to establish tumor cell cultures, conditional reprogramming (CR), with a rapid, reproducible and robust *in-vivo* zebrafish model. We have established cell cultures from two individual ACC PDX tumors that maintain the characteristic *MYB* translocation. Additional mutations found in one ACC culture also seen in the PDX tumor. Finally, the CR/zebrafish model mirrors the PDX mouse model and identifies regorafenib as a potential therapeutic drug to treat this cancer type that mimic the drug sensitivity profile in PDX model, further confirming the unique advantages of multiplex system.

## Introduction

Adenoid cystic carcinoma is relatively rare salivary gland tumor that frequently arises in young to middle aged adults. Despite its low incidence, it has a lengthy clinical course, hence a disproportionate disease burden. Though slow growing, it has the propensity for early invasion of peripheral nerves or blood vessels, resulting in a high incidence of local recurrence and distant metastases (e.g. lung and bone)^1–3^. The primary course of treatment is surgical excision combined with postoperative radiotherapy, but there is no known effective therapy for metastatic disease. Though *MYB* mutational activation is known to occur in the majority of ACC (Persson *et al*., 2012), little else is known about the downstream consequences of MYB activation and other molecular factors involved in the initiation and progression of ACC due to a lack of stable cell cultures. Recently, patient-derived mouse xenografts (PDX) have been successfully established from ACC primary tumors. These PDX tumors have been shown to maintain the histology and gene expression profile of the primary tumor, making them a valid model system for drug discovery^4, 5^. However, PDX models suffer from high cost, extended time required for tumor generation, low take rate (30–50%), and lack of manipulation and high throughput capability^6^. Additionally, repeated passaging of PDX tumors often results in the evolution of tumor histology and cell signaling pathways. Finally, it is not possible to generate normal epithelial cells from the same patient using PDX models. Access to both normal and cancer cell cultures, especially when derived from the same patient, is very useful for comparative drug screening and for efforts towards personalized medicine. Recently, several distinctive molecular features of ACC have been reported, including that a majority of ACC have a translocation of chromosome 6, resulting in fusion of the *MYB* gene with *NFIB* located on chromosome 9^7–11^. In addition, gene expression profiling has identified activation of TrkC signaling and other pathways^12–15^. However, the biological significance of these and other molecular attributes of ACCs are unknown due to the lack of stable cell cultures in which to perform experimental interrogation.

One of the greatest challenges in cancer biology research is the development of a method to generate stable cancer cell cultures from primary tumors that retain their specific phenotypic characteristics and genetic background. Interestingly, while PDX models of ACC have been generated, there are no ACC cell cultures that have been validated to mimic the genotype of the parent tumor. The few cell cultures that have been described in the literature lack the characteristic *MYB-NFIB* translocation and/or expression of MYB protein^16, 17^. In addition, several of these cultures are contaminated with other cell lines such as HeLa^18^. A new cell culture method recently described by our lab (conditional reprogramming, CR) combines the use of irradiated mouse fibroblasts and a Rho-associated protein kinase (ROCK) inhibitor to efficiently generate cell cultures. The CR method can produce long-term cultures from both normal and cancer tissues without using additional immortalization techniques. These cells have been shown to maintain a karyotype similar to the tissue of origin, even after prolonged passaging^19–22^. In this report, we have established two individual ACC cell cultures from PDX tumors using modified CR culture media conditions. We have also developed a rapid *in-vivo* zebrafish assay to validate the metastatic potential of the cultured tumor cells.

We examined one of the cell cultures (ACC11) for genetic alterations, protein expression and biological activity to evaluate whether it retained the key features of the tumor of origin. Additionally, we have used two independent ACC cell line models for regorafenib drug sensitivity and comparison with *in-vivo* models. This identified regorafenib as a potential therapeutic drug to treat ACCs. These models now provide the foundation for basic and translational studies, including the definition of the drivers of malignancy in this aggressive tumor.

## Results

### Establishment of ACC cultures

**Figure 1:**
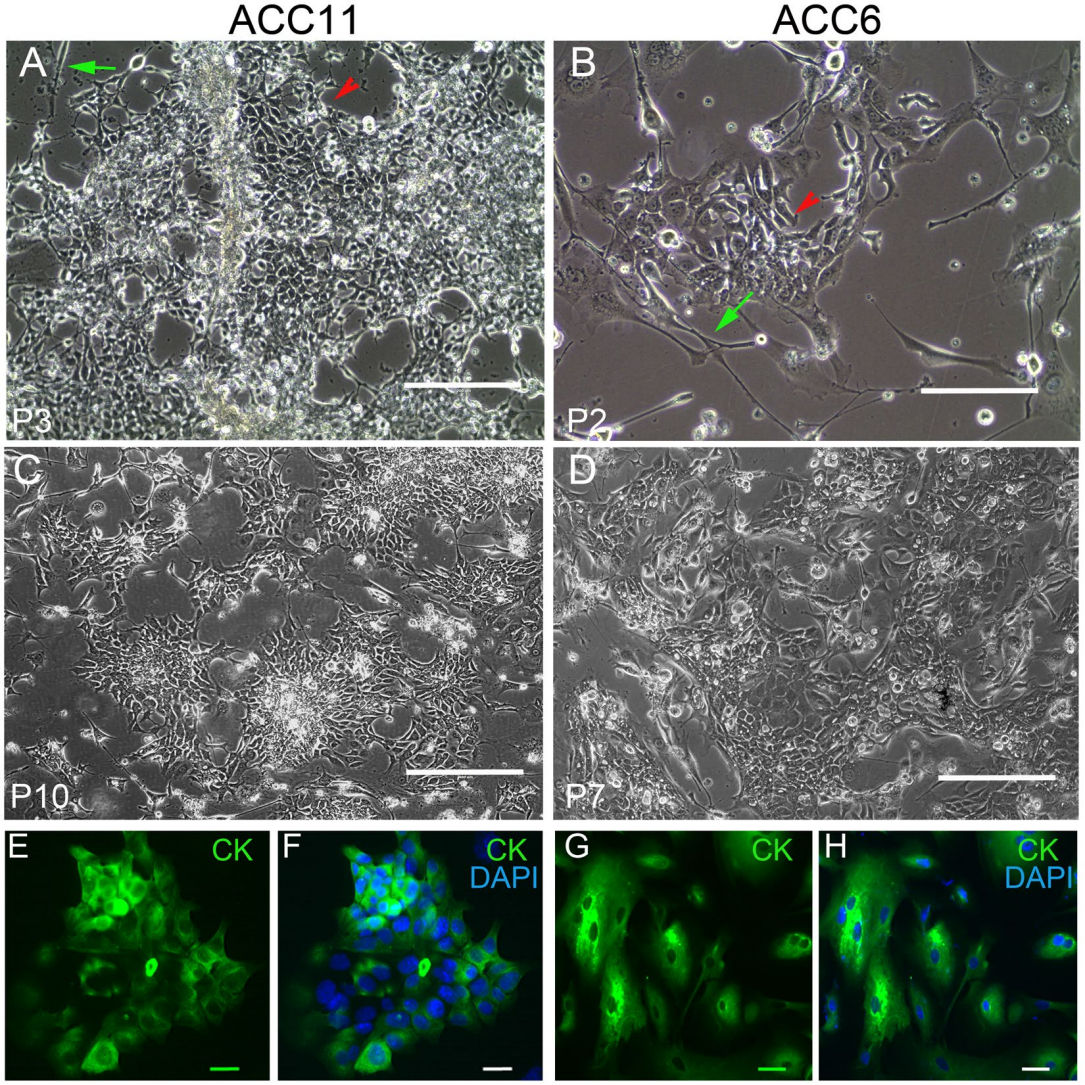
Morphology of ACC cell cultures and expression of epithelial cell marker. ACC11 and ACC6 cell cultures were established in 2D using CRC conditions. No obvious morphological changes were observed at different passages of cell cultures for both ACC11 (**A**,**C**) and ACC6 (**B**,**D**). Red arrowhead points to the epithelial tumor cells. Green arrows indicate irradiated mouse J2 cells. Magnification: 10x and scale bar: 200 μM. E-H: ACC11 (**E**,**F**) and ACC6 (**G**,**H**) cells were grown on glass coverslips and stained with pan-cytokeratin antibody to confirm the presence of epithelial cells and DAPI to visualize the nuclei. (**E**,**G**) pan cytokeratin (CK) expression; and (**F**,**H**): merged images for CK and DAPI. Magnification: 40x and scale bar: 10 μM.

Established PDX tissue materials were used to generate 2D cultures of ACCs. As described in the Methods section, tissue was minced and digested and plated in a modified CR medium with irradiated mouse fibroblast to establish stable cultures from two individual cases (Fig. 1A,B). These cell cultures were maintained only for limited passages (<15) and no obvious morphological changes were observed during passaging of these cells as shown in Figure C-D. Additionally, cytokeratin expression in both cell cultures indicates the epithelial nature of these cells (Fig. 1E–H).

### Short tandem repeat (STR) profiling

While there have been several reports describing the establishment of ACC cell cultures, detailed investigation has revealed that they all suffered from contamination with well-established cell lines. To validate the unique nature of cell cultures, we performed Short Tandem Repeat (STR) DNA fingerprinting. As shown in Table 1, both PDX tissue material and the corresponding cell cultures have an identical STR pattern which shows no similarity to cell lines in the ATCC database.

**Table 1:** STR data on ACC cell cultures and original PDX tissues.

Tissue/Cell Line	D8S1179	D21S11	D7S820	CSF1PO	D3S1358	TH01	D13S317	D16S539	vWA	TPOX	D18S51	AMEL	D5S818	FGA
ACCX11	13, 15	29, 31.2	8, 9	10, 12	15, 17	6, 9.3	11, 12	11, 12	17, 19	8, 10	12, 19	X	12	22, 24
ACC11	13, 15	29, 31.2	8, 9	10, 12	15, 17	6, 9.3	11, 12	11, 12	17, 19	8	12, 19	X	12	22, 24
ACCX6	10, 13	29, 31	11	11, 12	15, 16	9.3	9, 12	11, 12	15, 17	8, 9	12, 15	X	11, 12	21, 23
ACC6	10, 13	29, 31	11	11, 12	15, 16	9.3	9, 12	11, 12	15, 17	8, 9	12, 15	X	11, 12	21, 23

ACCX11 and ACCX6 represent PDX tissues and ACC11 and ACC6 are PDX-derived cell cultures.

### Genomic and molecular characterization

#### Chromosomal validation of MYB translocation in ACC cell cultures

**Figure 2:**
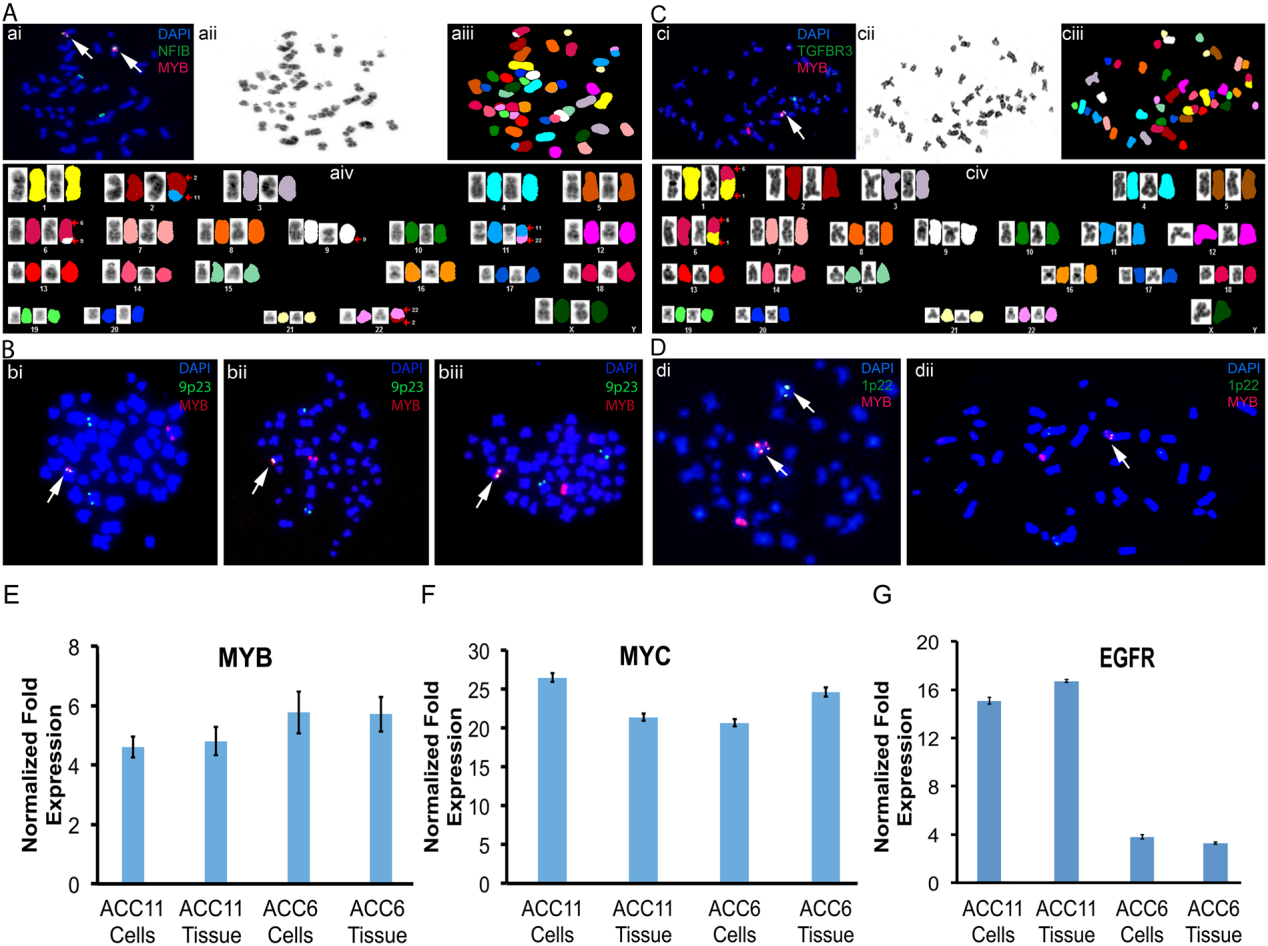
*MYB* gene translocation and *MYB*, *MYC* and *EGFR* gene expressions are maintained in ACC cells. (**A**) SKY analysis of ACC11 metaphase spread showed 6:9 translocation confirming the *MYB-NFIB* translocation (aiii and aiv). In addition, a few novel translocations were discovered, red arrows in aiv. The same metaphase, as in aiii, was used for FISH analysis using probes specific for *MYB* (red) and *NFIB* (green) genes (ai). (**B**) Metaphase nuclei from ACC11 cells were stained with FISH probes for *MYB* (red) and chromosome 9p23 (green) to confirm the results from Aai. All three individual nuclei show co-localization of *MYB* and chromosome 9p23 (white arrows). At least 25 nuclei were counted for each FISH experiment and DAPI was used to visualize chromosomes. (**C**) SKY analysis of ACC6 metaphase spread showed *MYB-TGFBR3* translocation (ciii and civ) and the same metaphase (ciii) was used for FISH analysis using probes for *MYB* (red) and *TGFBR3* (green) to show co-localization of these probes (white arrow) (ci). (**D**) Metaphase nuclei from ACC6 cells were stained with FISH probes for *MYB* (red) and chromosome 1p22 (green) to confirm the results from Cci. The white arrow points to *MYB* translocation. (E–G) *MYB* (**E**); *MYC* (**F**); and *EGFR* (**G**) gene expression levels in ACC11 and ACC6 cell cultures were compared with their corresponding PDX tissues. *GAPDH* gene expression was used to calculate and normalize the ΔCt values.

The ACC11 culture was first assessed for the *MYB* translocation at the DNA level. Fluorescence *in situ* hybridization (FISH) was performed on ACC11 metaphase nuclei using a combination of *MYB* and *NFIB* probes (Fig. 2Aai), and further confirmed by using *MYB* and chromosome 9p23 probes (Fig. 2B). A total of twenty-five metaphase nuclei were counted for each probe pair (*MYB* and *NFIB* or *MYB* and chromosome 9p23). The *MYB-NFIB* translocation was observed in 100% of the metaphase preparations, suggesting that it is likely an early event in the progression of this cancer^9^.

For the ACC6 PDX model, the *MYB-TGFBR3* translocation was reported previously^11^. Thus, the *MYB* translocation in ACC6 cell culture was confirmed by using *MYB* FISH probe combined with a Chromosome 1p22 probe and separately *MYB* FISH probe combined with a *TGFBR3* specific FISH probe (Fig. 2Cci,D). This translocation was present in all metaphases suggesting that *MYB* translocation was likely to be an early event in tumor evolution.

Spectral karyotyping (SKY) analysis was performed on the same nuclei used for FISH in order to further confirm *MYB* translocation in both ACC cell cultures. For ACC11, SKY revealed new translocations along with the 6:9 translocation for *MYB-NFIB* that were not known for this tumor and were also present in all metaphase nuclei. The translocations are 44–46,XX,t(2;11)(q37;q13), der(6)t(6;9)(q23;p23), t(11;22)(q13;q12) and der(22)t(2;22)(q37;q12) (Fig. 2Aaiv). In contrast to our findings in ACC11 cells, SKY analysis of the ACC6 cell culture showed no additional translocations beyond *MYB-TGFBR3* (Fig. 2Cciv).

#### RNA evidence for MYB-NFIB translocation

**Figure 3:**
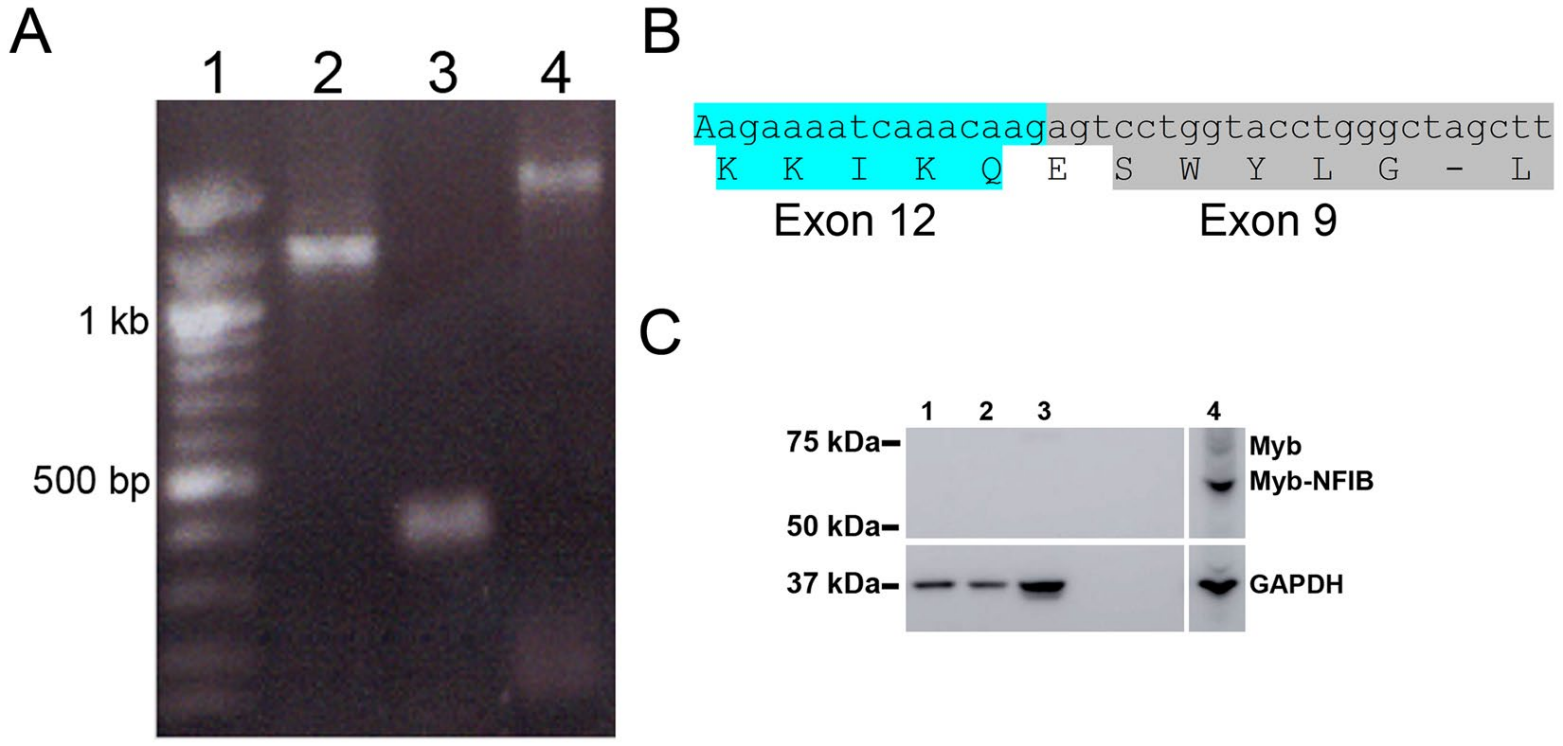
*MYB-NFIB* transcript and fusion protein are preserved in ACC11 cells. (**A**) *MYB-NFIB* transcript is present in ACC11 cells. Primers specific to *MYB* (exons 6, 14 or 5 in lanes 2, 3, and 4, respectively) and *NFIB* exon 9 were used for RT-PCR analysis of ACC11 RNA. *MYB* exon 14 specific primers failed to yield the expected PCR product of ~200 bp (lane 3), while *MYB* exon 6 and *MYB* exon 5 specific primers produced the predicted size products of ~1.2 and ~1.4 kb respectively (lanes 2 and 4, respectively). Lane 1: 100 bp DNA ladder. (**B**) Partial sequence result of the *MYB* exon 6 – *NFIB* exon 9 PCR product from lane 2 of the panel A to show the breakpoint for ACC11. The *MYB* breakpoint is in exon 12 as shown and NFIB breakpoint is in exon 9 as shown for ACC11 cells. *MYB* sequence (RNA and corresponding amino acid sequence for exon 12) at the fusion is highlighted in blue, while NFIB sequence is highlighted in gray color for exon 9. (**C**) Myb protein including Myb-NFIB fusion protein is overexpressed in ACC11 cells as compared to non-ACC cell cultures. Western blot analysis of cell extracts for Myb protein (top panel), and GAPDH was used as a protein loading control (bottom panel). Lane 1: BN (breast normal CR cells); 2: MCF7; 3: MDA-MB231; 4: ACC11. Lane 4 is a separate lane as one of the lane between lane 3 and 4 was cropped out from the original gel blot.

Alternative splicing and variable breakpoints in *MYB* and *NFIB* have been reported in ACC^7, 9, 23–25^. Thus, we checked the presence of *MYB-NFIB* fusion transcript in ACC11 cell cultures by performing RT-PCR using primers specific for *MYB* (exon 5, 6 or 14) and *NFIB* (exon 9). We detected a defined RT-PCR product of ~1.4 kb or ~1.2 kb when using primers specific for exon 5 or 6 respectively (Fig. 3A, lane 4 and 2). Based on the PCR product sizes observed for exon 5 or 6 for *MYB*, we predicted the size to be of ~200 bp PCR product with exon 14 for *MYB*. However, we observed a ~400 bp product (Fig. 3A, lane 3). This suggested that the breakpoint for *MYB* was prior to exon 14. Sequencing of gel purified PCR bands from Lanes 2 and 4 from Fig. 3A with corresponding *MYB* and *NFIB* primers identified the breakpoint for *MYB* at the end of exon 12 and for *NFIB* at the start of exon 9 (Fig. 3B), which encodes for only last four amino acids for *NFIB*. These breakpoints have been reported previously. This was further confirmed by sequencing the RT-PCR product from the starting PDX tumor (data not shown), showing the faithful maintenance of the translocation and fusion product, which is lost in other previously reported ACC cell cultures^17^.

#### Myb-NFIB fusion protein is overexpressed in the ACC11 cell culture

Previous ACC cultures have lacked Myb protein overexpression presumably due to a failure to maintain the *MYB-NFIB* translocation. Myb overexpression is the hallmark of ACCs, thus loss of this protein expression in previous cell cultures is a serious impediment to studying the biology of this tumor. Since our cultures maintained the translocation, we predicted that they might also overexpress Myb proteins. In ACC11 cells the predictive size of the fusion protein is 534 amino acids as compared to 640 amino acids for the wild type Myb protein. As shown in Fig. 3C, (lanes 4), Myb fusion protein overexpression was maintained in the ACC11 cell culture. As expected, the predominant band was smaller (~60 kDa) than the full-length Myb protein (~70 kDa). The full-length Myb protein was present at much lower level. In contrast to ACC11 cells, Myb protein was not detected in non-ACC cell cultures/lines (lanes 1–3).

#### Gene expression levels of MYB, MYC and EGFR are maintained in cell cultures

qRT-PCR was performed to compare the RNA levels of *MYB* in both cultures with their corresponding PDX tissue material (relative to endogenous *GAPDH* mRNA). No discernible differences in the transcript levels were observed between cell culture and corresponding PDX tissue material indicating faithful maintenance of the gene expression levels for the key gene as shown in Fig. 2E. *MYC* is a well-defined downstream target for *MYB*, thus we examined the gene expression levels of *MYC* in our cultures and compared it with their corresponding tissues. Additionally, we determined and compared the level of *EGFR* gene expression in both cell cultures and their corresponding PDX tissue material. As shown in Fig. 2F,G, we observed similar levels of gene expression for *MYC* and EGFR genes.

#### Mutations in key cancer genes are maintained in ACC11 cell culture

**Figure 4:**
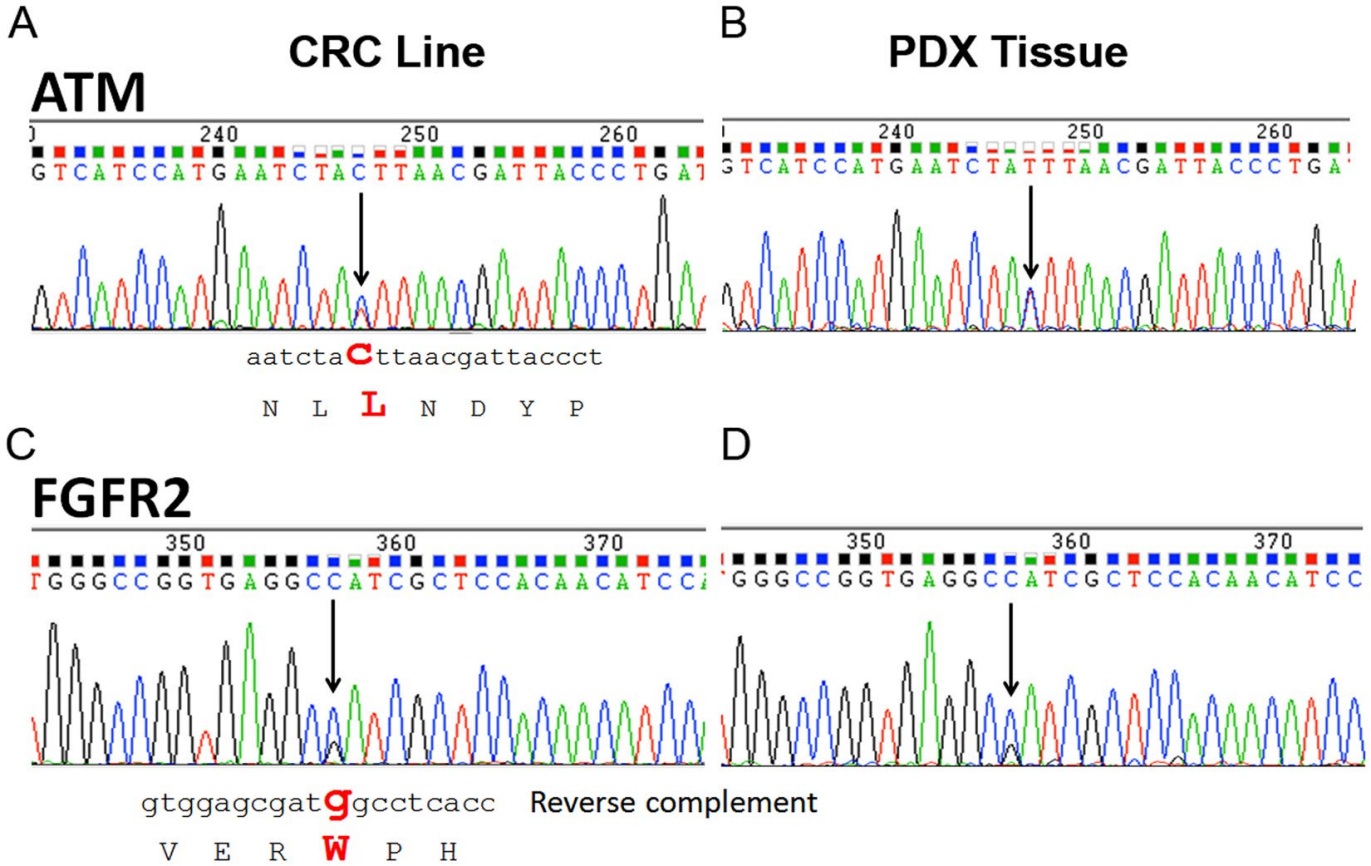
ACC11 cell culture and its parent PDX tumor express mutated *ATM* and *FGFR2* transcripts. Sequencing of *ATM* transcript confirms the point mutation at 2572 T > C generating a F858L point mutation in the ACC11 cell culture (**A**) and in PDX tissue (**B**). The *FGFR2* transcript shows a point mutation at 755 C > G resulting in non-synonymous oncogenic mutation of S252W for ACC11 cells (**C**) and PDX tissue (**D**).

Using next generation sequencing for the 48 TruSeq cancer panel, we identified oncogenic mutations in *FGFR2* and *ATM* genes in the ACC11 cell culture that had not been reported for this tumor. A non-synonymous point mutation in the *ATM* gene (2572 T > C) was identified resulting in a F858L mutation. Similarly, the *FGFR2* gene showed a heterozygous point mutation at 755 C > G resulting in a non-synonymous mutation of S252W. We validated the presence of these mutations at the transcript level by sequencing *ATM* and *FGFR2* specific RT-PCR products respectively (Fig. 4A,C). These mutations were further confirmed in the RNA of PDX tissue material where wild-type (WT) and mutated transcripts were present in equal amounts as observed in the cell culture (Fig. 4B,D). This indicates that the ACC11 cell culture has maintained the mutations and transcript levels as in the PDX tissue material.

Mutations of the *FGFR2* and *ATM* genes that are found in ACC11 are also found in other tumors especially in a subset of endometrial cancers and sporadically in other cancer types^26-30^. Actually, the FGFR family of genes has been identified as the most frequently mutated and/or amplified in ACC. Based on the data from these genetic studies, FGFR2 has been identified as an important target in ACC, but thus far, no studies have been done to functionally validate this as a molecular driver and/or a drug target^31–33^. The role of *ATM* mutation in tumorigenesis is not well defined, but it has been reported for several cancers^34–36^. The current cell culture model system will provide a unique opportunity to understand the role of the FGFR family and how they may interplay with Myb overexpression in ACC.

### ACC11 cells maintain a transformed and invasive phenotype

#### Soft agar assay

**Figure 5:**
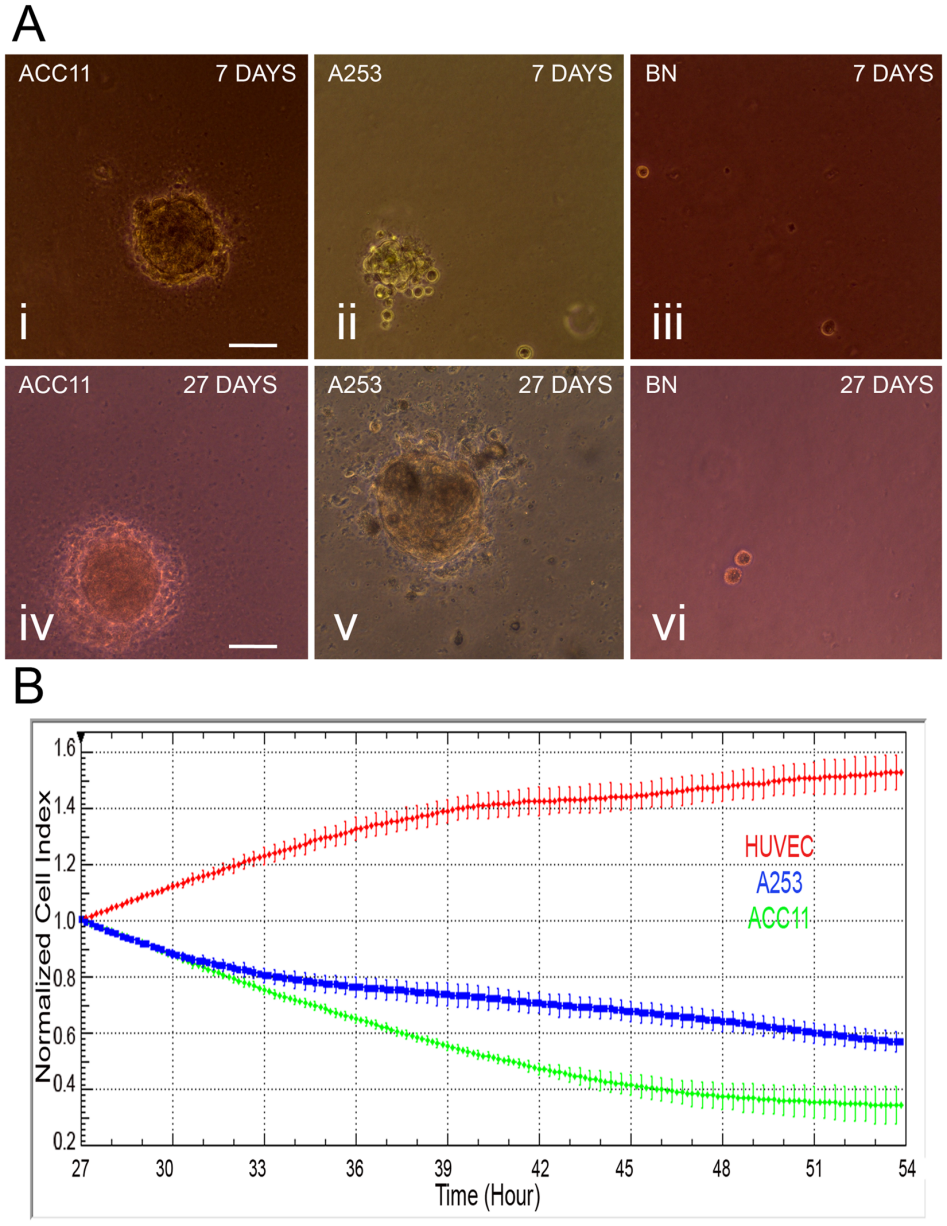
ACC11 cell culture maintains transformed and invasive properties. (**A**) ACC11 cells are transformed as shown by colony formation in an anchorage independent soft agar assay. ACC11 cells make microscopic colonies as early as 7 days (Ai, iv). A253 cells are used as a positive control showing colony formation (Aii, v) and BN (breast normal CR) cells were used as a negative control (Aiii, vi). Magnification: 20x and scale bar: 100 μM. (**B**) ACC11 cells (green) invade a monolayer of HUVEC cells as measured by electric impedance recorded by the xCELLigence RTCA SP instrument. A253 cells (blue) are used as a positive control. Cell media was used as a baseline control for HUVEC cells (red). Each cell culture is used in six replicates and data is shown as a normalized and averaged cell index. Tumor cells were added on the top of the HUVEC monolayer once it is formed in each well.

The formation of colonies in soft agar reflects the ability of tumor cells to grow and divide independent of substrate attachment (an anchorage independent growth), a characteristic of tumor cells^37^. We tested the potential of ACC11 cells for their transformative potential in soft agar. The ACC11 cells formed only microscopic colonies in soft agar as early as 7-days, but did not form macroscopic colonies even at 27 days (Fig. 5Ai,iv). In comparison, commercially available A253 cells (a mucoepidermoid tumor cell line of the salivary gland) showed both microscopic and macroscopic colonies in soft agar at early (7 days) and late time points (27 days) respectively (Fig. 5Aii,v). A breast normal CR cell line (BN) was used as a negative control and as expected it did not show any colony formation even at 27 days (Fig. 5Aiii,vi).

#### Invasion assay

The ability of tumor cells to invade a monolayer of endothelial cells *in-vitro* is used as a surrogate marker for their *in-vivo* biological invasiveness. We investigated the potential of ACC11 cells to invade a monolayer of HUVEC cells as measured by electric impedance. A253 tumor cells were used as a positive control. As shown in Fig. 5B, both A253 and ACC11 cells decreased the electric impedance of HUVEC cells, indicating invasion of the endothelial cell barrier.

### Establishment of a rapid, robust and reproducible zebrafish in-vivo tumor metastasis (ZTM) model system

**Figure 6:**
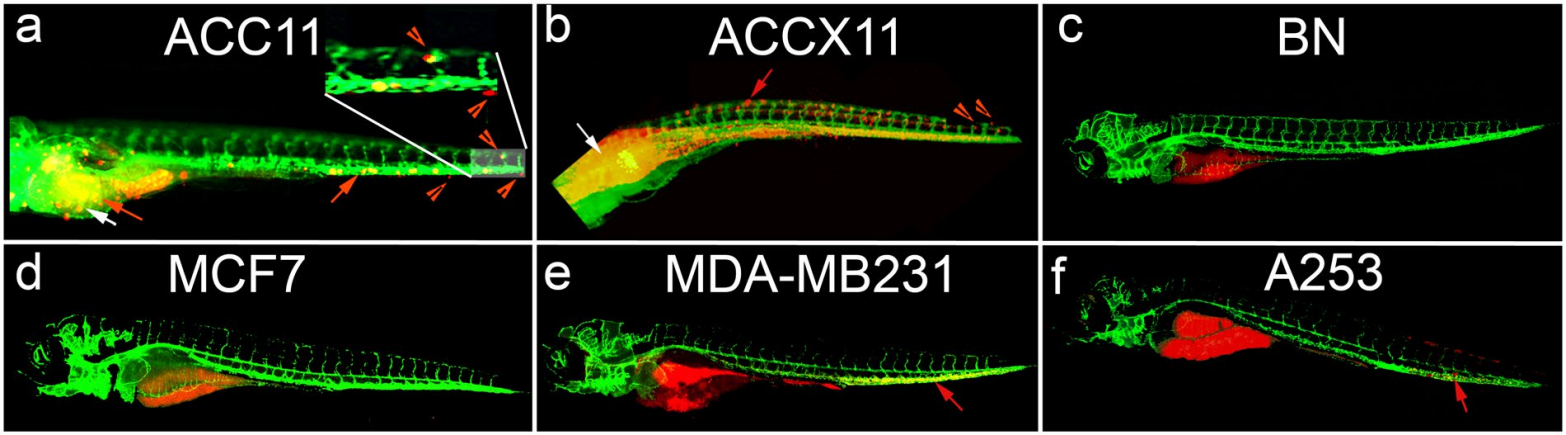
ACC11 cells show metastatic potential in an *in-vivo* zebrafish tumor metastasis model system. ACC11 cells (**a**), ACC11 PDX tissue (**b**), normal breast CRC cells (**c**), MCF7 cells (**d**), MDA-MB-231 cells (**e**), or A253 cells (**f**) labeled with CM-dil (red) were injected into the yolk sack of 2 day post fertilization (dpf) zebrafish embryos, and then imaged 3–7 days post injection. The vasculature is labeled green. White arrows indicate the area of injection in the yolk sac and red arrows indicate migrated ACC11 cells in the yolk sac in the main vessel (migration and metastasis), while red arrowheads indicate the cells that have extravasated from the main vessel into neighboring caudal hematopoietic tissue. Higher magnification for the tail region in panel (a) as an inset to show the extravasation of ACC11 cells into the neighboring tissue.

The mouse xenograft model has been established for ACCs, but it has major drawbacks including very slow initiation (it takes 2–12 months to establish a xenograft), lacks high-throughput capability, is expensive, has a low success rate of approximately 30–60%, and cannot interrogate the metastatic potential of tumor cells. In contrast, the ZTM model has all the advantages that above-mentioned mouse model lacks^38–42^. Injection of ACC11 cells into the yolk sack of 2 day post-fertilization stage (2dpf) embryos resulted in rapid movement of a small fraction of cells to the tail and head regions as early as 3 days post injection demonstrating the metastatic potential of ACC11 cells (Fig. 6a). Cells have not only intravasated the main blood vessel (red arrows in Fig. 6), but also a few cells extravasated into the neighboring tissue demonstrating the metastatic process (red arrowheads in Fig. 6a and inset for higher magnification image). Similar results were obtained when a small piece of cryo-preserved PDX tissue material was transplanted into 2 dpf zebrafish embryos (Fig. 6b), thus the ACC11 cells have maintained the metastatic potential of the primary tumor. In the case of PDX tissue, it took 6 days for the cells to show invasion and extravasation of tumor cells. This is not surprising as the tumor cells are within an *in-vivo* tumor architecture along with stromal cells that most likely interferes with early invasion. In order to ensure that the adult stem cell-like property of all CR cells is not the reason for the metastatic behavior of ACC11 cells, we injected normal CR breast cells and they did not show migratory behavior (Fig. 6c). Similarly, a non-metastatic breast cancer cell line, MCF7 did not show metastatic potential (Fig. 6d) even after 7 days post injection. Finally, a highly metastatic breast cancer cell line, MDA-MB231, and A253 cells showed metastatic properties analogous to the ACC11 cell culture (Fig. 6e,f).

### *In-vitro* and *in-vivo* drug sensitivity assay for ACC cultures

#### *In-vitro* assay

**Figure 7:**
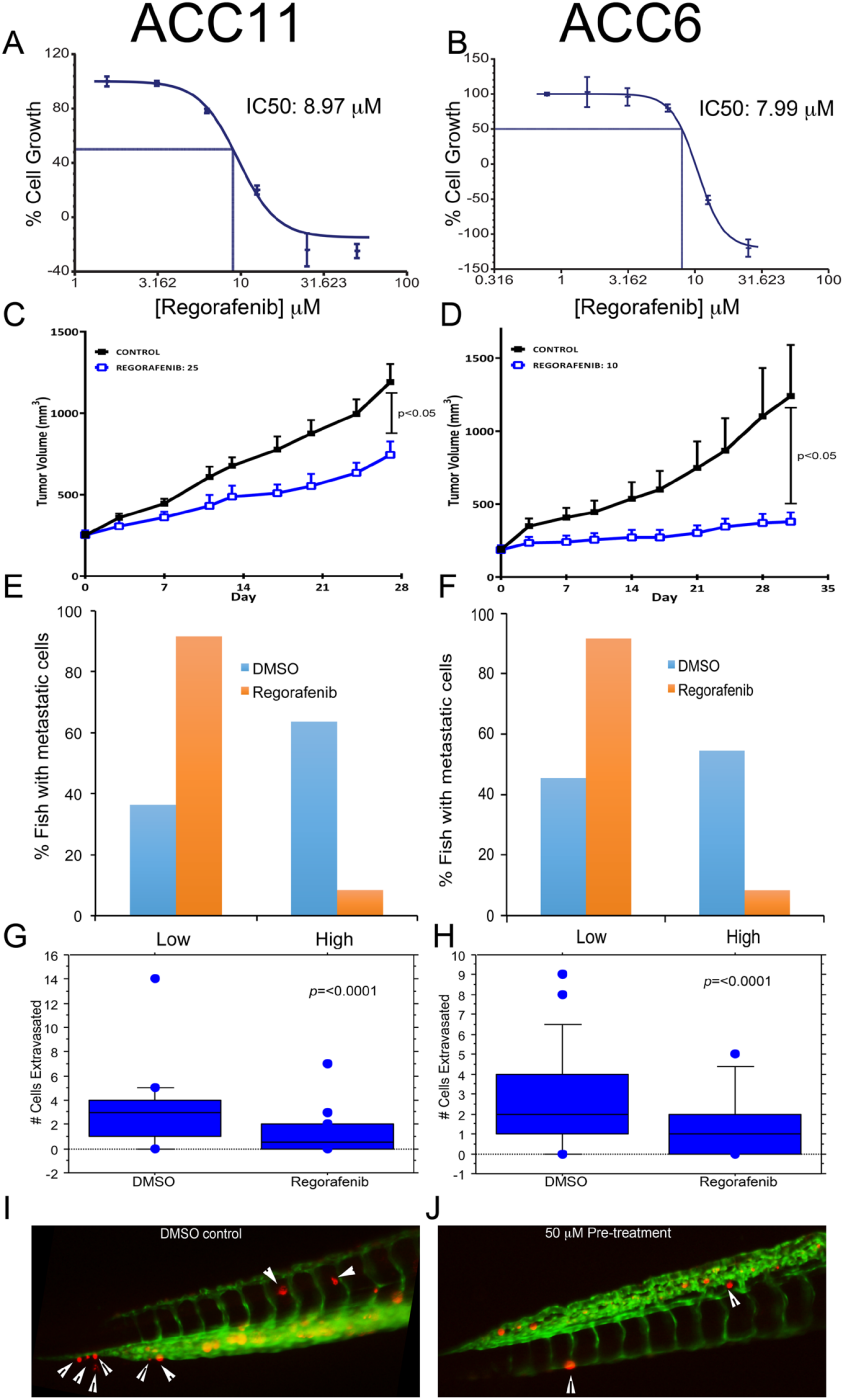
ACC11 and ACC6 cells show similar drug sensitivity in cell-based and in zebrafish assays when compared to corresponding mouse xenograft drug sensitivity. A-B: Cell-based drug sensitivity assays for regorafenib for both ACC11 (**A**) and ACC6 (**B**) cells using Incucyte. (**C**,**D**) Regorafenib sensitivity of ACC11 (**C**) and ACC6 (**D**) in PDX model. E-F: Labeled ACC11 PDX tissue material (**E**) and ACC6 cells (**F**) were injected into the 2dpf zebrafish embryos and were arrayed in 96-well plate with continuous exposure to 0.3 μM of regorafenib or 1% DMSO (control group) in ZTM assay. Cells migrated to the tail after 5 days were scored in both groups. Fish with 0–3 cells were grouped as low and ≥ 4 cells migrated to the tail were grouped as high. Percentage fish with low and high metastatic cells for both treated and un-treated were plotted in excel. G-H: ACC11 cells (**G**) and ACC6 cells (**H**) were pre-treated with 50 μM regorafenib or 1% DMSO for 45 minutes prior to the injection into the yolk sinus in zebrafish extravasation assay. Fish were arrayed in 96-well plate and all were treated with 0.3 μM of regorafenib. Extravasation was scored in 24 hours for ACC11 (**G**) and 48 hours for ACC6 (**H**) cells. The results are plotted as box plots for each cell line. (**I**,**J**) Representative images for extravasated red labeled ACC11 cells in DMSO control group (**I**) and in 50 μM pre-treated group (**J**). White arrowhead points to the extravasated cells in the caudal region of the tail.

The drug screening capability of ACC cultures was assessed using two different cultures and a drug that is currently being investigated for its clinical potentials in Head & Neck and other tumor types^43, 44^. Regorafenib is an FDA approved multi-kinase small molecule inhibitor that targets VEGFR2 and TIE2 tyrosine kinase receptors (RTK) and shows anti-angiogenic activity in clinical settings for the treatment of gastrointestinal and colorectal cancers. However, this drug also inhibits other RTKs at much higher doses. Thus, we decided to test regorafenib in ACC11 and ACC6 cell line models by exposing the cells to different concentrations of regorafenib in 96-well format and the cell proliferation was monitored using an Incucyte high content imager for 72 hours. Cell confluency was used as a measure of cell proliferation and was quantified by the analysis software. ACC6 cells (IC_50_: 7.99 μM) were slightly more sensitive to regorafenib compared to ACC11 cells (IC_50_: 8.97 μM) in this assay (Fig. 7A,B). These IC_50_ values are comparable to the clinical exposure (C_max_ 3.9 μg/ml equivalent to 8.09 μM) to this drug^45–47^.

#### *In-vivo* assays

*Mouse xenograft for drug screening*: Preclinical studies were conducted at START (South Texas Accelerated Research Therapeutics, San Antonio, TX, USA) under International Animal Care and Use Committee-approved protocols by START. Briefly, ACC11 and ACC6 tumor fragments were harvested and implanted subcutaneously into the flank of athymic nude mice (Charles River Laboratories). On Day 0 animals were randomized to control (C) and treatment (T) groups and the study initiated at a mean tumor volume (TV) of approximately 225 mm^3^. Tumor volume and animal weight data were collected electronically using a digital caliper and scale; tumor dimensions were converted to volume using the formula TV (mm^3^) = width^2^ (mm^2^) × length (mm) × 0.52. Tumor growth inhibition (TGI) was defined as the ratio of geometric mean tumor volume of treated group compared with vehicle treated group with corresponding 95% confidence intervals for comparisons. In the ACC11 study, animals were randomized into untreated control (n = 7) and treatment (n = 4) groups that were administered 25 mg/kg regorafenib. The study was ended on Day 27 with a calculated %T/C of 42 with TGI of 48%(Fig. 7C). In the ACC6 study, animals were randomized into untreated control (n = 9) and two treatment (n = 5 for each group) groups that were administered 10 mg/kg or 30 mg/kg regorafenib. The study ended on Day 31 with a calculated %T/C of 16 with TGI of 77% and showed no difference in tumor growth suppression between the two doses (Fig. 7D and data not shown). Regorafenib was well tolerated and did not cause significant weight loss or any other overt clinical symptoms. Regorafenib led to statistically significant delay in the tumor growth in both model systems (P < 0.05), but showed better efficacy in the ACC6 model compared to the ACC11 model similar to the results for *in-vitro* studies. Our data suggests that regorafenib as a potential therapeutic drug to treat ACCs. Both, ACC11 and ACC6 have *MYB* gene translocation in common, thus in the future it would be interesting to further explore the potential of this drug for the treatment of *MYB* translocated ACCs.

#### Zebrafish ZTM and extravasation assays for drug screening

To further confirm our cells and mouse PDX results and validate the zebrafish ZTM and extravasation assays for drug screening, ACC11 PDX tissue and ACC6 CR cells were injected into the yolk sac of 2dpf zebrafish embryos. Zebrafish were arrayed in 96 well plates and treated with the maximum tolerated dose (MTD) of regorafenib for 4 days. As a measure of metastatic potential, the number of embryos with cells that had migrated to the tail were scored after 4 days. The number of cells migrated to the tail were grouped into two categories, low group where only 0–3 cells migrated and high group where ≥ 4 cells were migrated to the tail region. Regorafenib treatment disrupted the migration and intravasation of both ACC11 PDX tissue and ACC6 cells compared to the vehicle treated control groups. This inhibition was irrespective of whether PDX tissue or CR cells were used (Fig. 7E,F), again validating that the cultured cells mimicked the biology of the tumor.

To further analyze the metastatic potential of tumor cells *in-vivo*, we determined the potential of these tumor cells to extravasate from the blood vessels into the neighboring tissue. For this, labeled cells were directly injected into the pre-cardiac (yolk) sinus of 2 dpf embryos allowing cells to distribute throughout the vascular system^40, 48, 49^. Extravasation was scored in the tail region one to three days following injection. The tail region was scored because this tissue is flat and a majority of extravasated cells tend to localize to the caudal hematopoietic tissue in the tail. This technique is a rapid and robust way to assess the metastatic potential of cells using extravasation as a surrogate marker. In this system, we found that both ACC11 and ACC6 cells rapidly extravasated by 24 and 48 hours post injection, respectively. Furthermore, pre-treatment of ACC11 and ACC6 cells with 50 μM of regorafenib for 45 minutes prior to injection followed by continuous treatment of embryos with MTD of drug (0.3 μM) led to reduction in the metastatic potential of tumor cells as shown in the Fig. 7G–J. In the control group most fish had > 3 cells extravasated, while in the drug treatment group showed mostly 0–2 cells extravasated, as shown using box plots in Fig. 7G,H. Paired student t-test indicated that the difference between pre-treatment vs untreated groups was statistically significant with the *p* value of < 0.0001 for both cell lines.

## Discussion

We have demonstrated that conditional reprogramming can be successfully used to establish well-authenticated cell cultures for ACCs that maintain the key molecular and cellular features of the tumor of origin. Drug sensitivity assays indicate that CR cells show similar drug sensitivity both in *in-vitro* (cell based) and *in-vivo* (zebrafish) assays and both of these assays correlate with the mouse PDX model. The use of CR-generated cultures will now allow the systematic basic and translational studies for this tumor that were not possible before.

The CR culture system can be used to generate both normal and ACC salivary gland cultures and it can be easily manipulated genetically for the systematic exploration of the basic biology and molecular drivers for ACC. While a few attempts have been already made to evaluate the biological consequence of expressing the mutated genes of ACC^11, 50^, these studies have used artificial recipient cell cultures such as NIH3T3 and Jurkat cells for oncogene expression. Such studies obviously do not reflect the biology of ACC cells. This problem can be partially overcome by using normal salivary gland cultures as the target for oncogene expression. Thus, single and multiple genetic alterations can be made in the normal salivary gland cells to mimic those changes found in the ACC tumor. Overexpression of *MYB* or transfection of the *MYB-NFIB* recombinant can be evaluated in normal salivary CR cells. In addition, it is now possible to study the effect of mutated *FGFR2* and *ATM* genes on normal salivary gland cells^31–33^. However, ACC cells may have additional genetic alterations that are critical for the full malignant phenotype. To address the potential role of other genetic alterations in ACC, malignant ACC11 and ACC6 cultures can be used for genetic knockdown or knockout experiments using siRNA, shRNA and/or CRISPR methods. In the future, the availability of having both normal and tumor cells from the same patient will provide a unique platform for genetic dissection of this aggressive cancer.

Finally, our SKY analysis has revealed novel translocations in ACC11 cells, and these additional chromosomal aberrations may have critical roles in the genesis of ACC. Future work will involve the validation of these translocations by FISH on PDX tumor tissue material, and the identification of the gene loci that are affected, and the respective role of these genes in ACC neoplasia.

Herein, we explored an *in-vivo* zebrafish tumor metastasis model system (ZTM) that works for tissue material as well as for cell cultures thereby making it an extremely useful model system to evaluate the tumor cell behavior and biology in the presence of its own stromal component. The zebrafish tumor models required very small amounts of tissue material so it is feasible to establish patient–derived zebrafish xenograft models and then to expand such grafts by CR. These zebrafish models could be used for a variety of applications, including metastatic potential of primary tumors in real time, genetic screening to identify molecular drivers for metastasis, drug screening, and to study potential tumor/stroma interaction important for tumor growth and metastasis^51–55^. Perhaps most important, these grafts can be established in 3–7 days rather than 3–7 months for the mouse model. Thus far, no other *in-vivo* model system exists that can provide the rapid, real-time investigation of metastasis (migration, intravasation and extravasation) and cost effective drug screening platform that works in a very short period of time (Bentley *et al*., 2015; Bulut *et al*., 2012).

ACC are different from many other solid cancer types in that they do not harbor many mutations, genetic translocations and/or copy number variations. Thus, ACC is a great model system to identify the molecular drivers for cancer and to understand the interplay between a handful of genes and pathways and how they collaborate in the progression and metastatic potential of this tumor type. Cell culture models provide an opportunity to explore this in a very systematic fashion. A high-throughput shRNA/CRISPR screen can be carried out to identify important genes and pathways that are important for tumor initiation, progression and metastasis. The zebrafish *in-vivo* tumor metastasis model and/or extravasation model systems can be used to verify the *in-vitro* data in a very rapid and reproducible fashion. The zebrafish models will help to segregate the molecular drivers that are important only for tumor initiation and progression, but not for metastasis and vice versa. This will be the first time a model is described which can be used with ease to test the migration, invasion and metastasis properties of tumors.

The lack of authenticated cell cultures for ACC has impeded basic research as well as drug target identification and screening. CR technology has changed this by providing a reliable, reproducible and authenticated cell culture model system. It is possible to generate cell cultures from fresh or cryo-preserved patient tissue and we anticipate that such cultures can be used successfully for high-throughput drug screening. High-throughput drug screening is impossible to carry out using mouse xenografts, but they are useful for validating a drug and its targets. Our *in-vivo* zebrafish tumor metastasis (ZTM) model system as well as extravasation model can be used in drug screening platforms as a first pass before testing in mouse xenografts that are still considered a gold standard. It remains to be seen whether regorafenib will be a useful drug to treat ACCs in a clinical setting. Further work is warranted to test regorafenib in additional *in-vitro* and *in-vivo* ACC models.

Currently we do not know whether the ACC11 and ACC6 cell cultures reflect the complete genotype and phenotype of the primary tumor or whether the primary tumor has significant heterogeneity and if heterogeneity is present in the ACC cell cultures. Cell cultures utilizing the patient’s primary tumor, along with detailed genomic analysis, should be able to address this issue. However, this tumor type is relatively rare and it will require nation-wide collaboration efforts to secure sufficient tumor specimens for analysis. On the positive side, our previous studies have shown that our approach has worked with another rare tumor (Neuroendocrine cervical cancer)^56^. We anticipate that CR will be able to generate many cell model systems, which are currently unavailable.

## Methods

All experiments involving human tissue and animal models were performed in accordance with relevant guidelines and regulations at Georgetown University, University of Virginia and/or START.

### Cultures for ACC

PDX tissues were obtained through the ACC Research Foundation (ACCRF) and Dr. Christopher Moskaluk’s lab where they were initially established. PDX tissues were minced, digested as previously described^19^ and plated in a modified CR cell media containing 1:3 ACC media and CR media. The CR media consists of 1:3 (v/v) F-12 Nutrient Mixture (Ham)–Dulbecco’s modified Eagle’s medium (Invitrogen, California, USA), 5% fetal bovine serum, 0.4 μg/mL hydrocortisone (Sigma-Aldrich, Missouri, USA), 5 μg/mL insulin (Sigma-Aldrich), 8.4 ng/mL cholera toxin (Sigma-Aldrich), 10 ng/mL epidermal growth factor (Invitrogen), 100 μg/ml Primocin (Invitrogen), and 10 μM Y-27632 (Enzo Life Sciences). The ACC media contains CR media with the following additional components: 100 ng/ml Noggin (Peprotech, New Jersey, USA), 3 μM SB202190 (Sigma-Aldrich, Missouri, USA), 20 ng/ml rhFGF (R&D Systems, Minnesota, USA), 3 μM CHIR-99021 (Selleckchem, Texas, USA), 20 ng/ml Wnt-3a (R&D Systems). Digested tissue was plated in this modified media with ~400,000 irradiated (40 Gy) J2 mouse fibroblast cells in a red cap T-25 flask (Greiner Bio One, VWR, PA, USA). All cultures were maintained in this media at 37 °C with 5% CO_2_ in a humidified chamber.

### Normal breast cell culture

Adjacent normal breast tissue was collected from a breast cancer patient at Georgetown University medical center with the informed consent of the patient according to a Georgetown University Hospital IRB protocol. The tissue was processed, digested and plated in CR medium in a T-25 flask (Greiner Bio-one, Sigma-Aldrich) with ~400,000 irradiated J2 mouse fibroblast cells and grown in a humidified chamber at 37 °C with 5% CO_2_ as previously described^19^. Normal breast (BN) culture was maintained under these conditions.

### STR profiling

Short Tandem Repeat (STR) profiling of DNA isolated from cultured cells or PDX tissue was performed by the Genetica DNA Laboratories, LabCorp, North Carolina, USA.

### MYB translocation

#### Spectral karyotyping (SKY) and Fluorescence in-situ hybridization (FISH)

Human metaphase chromosomes were prepared after incubation for 1–2 hours with 0.02 mg/ml Colcemid (Invitrogen; Grand Island, NY). The cells were then incubated in hypotonic solution and fixed with methanol/acetic acid (3:1). Metaphases were dropped onto slides using a Thermotron chamber to control for humidity. Spectral karyotyping (SKY) was performed using probes prepared in-house and analyzed as previously described (Schrock E *et al*., 1996). At least twenty-five metaphase nuclei were imaged and karyotyped using HiSKY software, version 7.2.7.31097. Characterizations of numerical and structural aberrations were described according to An International System for Human Cytogenetic Nomenclature (ISCN) 2013.

Fluorescence *in situ* hybridization (FISH) was performed using a standard protocol available under resources at https://ccr.cancer.gov/Genetics-Branch/thomas-ried. FISH analysis was performed using a FISH probe set targeting *MYB* (Empire Genomics; Buffalo, NY) with *NFIB* (Empire Genomics; New York, USA) or 9p23 for ACC11, or *MYB* with *TGFBR3* or Chromosome 1p22 probe (RPCI-11, clone 2B13 spanning region 92,384,975–92,554,252 of Hg19) in green (Empire Genomics) for ACC6. The probe corresponding to 9p23 was generated from RPCI-11 98J13, a bacterial artificial chromosome (Empire Genomics). At least twenty-five images were captured using a Leica DM-RXA fluorescence microscope (Leica; Wetzlar, Germany) equipped with a 40 X objective and custom optical filters. All slides were counterstained with 4′,6-diamidino-2-phenylindole to visualize chromosomes.

#### Immunofluorescence staining

Cells were grown on glass coverslips, fixed with 4% formaldehyde for 10 min followed by permeabilization with 0.2% Triton X-100 in PBS for 3 min. Coverslips were blocked for an hour in 2% BSA in PBS at room temperature followed by overnight incubation with anti-mouse pan-cytokeratin antibody (clone AE1/AE3, Dako, USA). On the second day, coverslips were incubated with goat anti-mouse Alexa 488 (ThermoFisher Scientific) for an hour at room temperature, followed by mounting of coverslips on glass slides with Prolong gold antifade reagent with DAPI (ThermoFisher). Images were captured at 40x magnification using Olympus PM-2000 microscope equipped with automated stage.

#### MYB-NFIB transcript and protein

RT-PCR was used to determine whether the *MYB-NFIB* fusion gene was expressed. Total RNA was isolated from cells and reverse transcription was performed using a random primer and Omniscript (Qiagen, Hilden, Germany) according to the instructions provided with the kit. *MYB-NFIB* fusions were amplified by PCR using total cDNA and *MYB* primers for exon 5 (5′ GGCAGAAATCGCAAAGCTAC 3′), exon 6 (5′ CTCCGCCTACAGCTCAACTC 3′), or exon 14 (5′ GCACCAGCATCAGAAGATGA 3′) paired with a *NFIB* exon 9 primer (5′ GTGCTGCAATTGCTGGTCTA 3′). The PCR products were gel purified and sequenced in both directions using *MYB* and *NFIB* primers.

For protein expression, total cell lysates were prepared using 300 μl of 2x Laemmli buffer to collect the ACC11 cells followed by heating at 95 °C for 10 min. Thirty micrograms of total protein was loaded on a 4–12% gradient Bis-Tris gel (Novex), transferred to a nitrocellulose membrane and probed with anti-Myb antibody (Abcam, Cambridge, UK). Myb protein levels were visualized using a chemiluminescent reagent (Pierce, Massachusetts, USA).

#### Real-time quantitative RT-PCR (qRT-PCR)

qRT-PCR was performed to measure the levels of gene expression of *MYB*, *MY*C and *EGFR* genes in cell cultures and compared it with their corresponding PDX tissue materials. Expression levels were normalized with *GAPDH* gene expression as described previously^57^. The following primers were used: MYB_R: 5′GGAGTTGAGCTGTAGGCGGAG; MYB_5 F: 5′ GGCAGAAATCGCAAAGCTAC;

MYC_F: 5′ACCACCAGCAGCGACTCTGA; MYC_R: 5′ TCCAGCAGAAGGTGATCCAGACT; EGFR_F: 5′ ACCTGCGTGAAGAAGTGTCC; EGFR_R: 5′ ATTCCGTTACACACTTTGCGGC;

GAPDH_F: 5′TCCCTGCCTCTACTGGCGCTGCCAAGGCTG; and GAPDH_R: 5′ TCCTTGGAGGCCATGTGGGCCATGAGGTCC. Normalized ΔCt values were calculated using formula: Ct_GAPDH_ − Ct_target_ for each experiment and normalized 2^ΔCt^ was plotted in excel. Each experiment was carried out in triplicates.

#### DNA and RNA sequencing

Total DNA was isolated from ACC11 cells using the DNA extraction kit for Animal Blood and Tissue from Qiagen. DNA was subjected to NGS for TruSeq cancer panel consisting of 48 key cancer genes by Genewiz, Massachusetts, USA, using Illumina’s MiSeq NGS platform. The mutated genes, *FGFR2* and *ATM*, were further confirmed for gene expression using total RNA. Primers close to the mutation sites were designed accordingly for both *FGFR2* (Forward primer: 5′ GCCAACCATGCGGTGGC 3′; Reverse primer: 5′CTATCTCCAGGTAGTCTGGG 3′) AND *ATM* (Forward primer: 5′ GACAAATGAGGAATTCAGAATTGG 3′ and Reverse primer: 5′ CGTACTCTTCTCCAGGAA 3′). RT-PCR followed by primer sets for each gene was used to amplify the mutated region and was subjected to sequencing using the PCR primers by Keck DNA sequencing core facility at Yale University, CT, USA.

#### Soft agar and Invasion assays

A soft agar assay was performed as described previously in a 12-well plate using CR media without fibroblast cells^58^. An invasion assay was done in a 96-well E plate that was coated with 0.1% collagen for an hour before plating 20,000 Human Umbilical Vein Endothelial Cells (HUVEC) cells in Endothelial Cell Growth Medium-2 (EGM-2) media (Lonza, Maryland, USA). The plate was left in the incubator for 16–20 hours to ensure the formation of a monolayer of HUVEC cells in each well as measured by a plateau reached for the electric impedance in the XCelligence RTCA SP instrument (ACEA Biosciences Inc, California, USA). Media was removed carefully from each well and replaced with CR media or media (McCoy’s 5 A medium supplemented with 10% FBS) specific for A253 cells. Ten thousand cells of ACC11 or A253 were plated in a 96 well using cell-specific media on the top of the monolayer of HUVECs for a total of six replicates for each. The plate was then left in the incubator for another 50 hrs. Normalized and averaged cell number indexes were calculated and the invasion was considered positive if the electric impedance was lost due to cells invading the HUVEC monolayer. Media alone was used as a negative control. Cells were considered negative for invasion if the electric impedance was maintained^59^.

#### Zebrafish *in-vivo* tumor metastasis model system

All animal procedures were conducted in accordance with NIH guidelines for the care and use of laboratory animals and approved all experimental protocols with zebrafish by the Georgetown University Institutional Animal Care and Use Committee. For the evaluation of metastasis, cells or tissue were first labeled with the lipophilic dye CM-diI (Thermo Fisher, V22885) according to the manufacturer’s instructions. Zebrafish embryos were injected with 100–200 labeled tumor cells or implanted (tissue) into the yolk sac at 2-day post fertilization (2dpf). We used transgenic zebrafish, *Tg(kdrl:grcfp)*, expressing green reef coral fluorescent protein in the vascular endothelium to enhance the tracking of tumor cell migration and invasion^40^. A minimum of thirty embryos were injected for each cell culture. Invasion of the vasculature was monitored as a surrogate of metastatic potential at 10x magnification using an Olympus IX-71 inverted microscope or a Zeiss LSM510/META/NLO laser scanning confocal microscope. Injected embryos were evaluated at 2–3 hour post injection to discard embryos from analysis if they showed any migration as that would be indicative of problems with the injection process. Embryos were evaluated daily for tumor cell migration and health of the embryos. For extravasation model, a minimum of 60 zebrafish were injected for each group in drug treatment assays.

### *In-vitro* and *in-vivo* drug treatment

#### *In-vitro* drug treatment

ACC11 (10,000 cells) or ACC6 (5,000) cells were plated in each well of a clear, flat bottom, 96-well plate in CR:ACC (3:1) media along with 1,000 irradiated feeder cells. Regorafenib treatment was done in various concentrations (3.125–50 μM range) in 1% DMSO in triplicate wells. Cells were incubated at 37 °C with 5% CO_2_ in a humidified chamber which is equipped with Incucyte high-content imager (Essen Bioscience, Michigan, USA). Images and percentage of cell confluency were collected every two hours for three days. The XLfit program (ID Business Solutions, Parsippany, NJ, USA) was used to obtain and calculate the IC_50_ curves for each compound using a non-linear regression curve fit utilizing Lavenburg–Marquardt algorithm. Each experiment was performed a minimum of three times.

### *In-vivo* drug treatment

#### Mouse model

All studies were performed under IACUC-approved protocols by STRAT and experiments were done at START. Serially-passaged xenograft tumor (PDX) fragments from host mice were harvested and implanted subcutaneously into immune-deficient mice and animals matched by tumor volume (TV) into control and treatment groups and dosing initiated. Tumor dimensions (mm) were converted to volume (mm^3^) using the formula: tumor volume = (width × 2) × length × 0.52. Regorafenib was formulated for daily oral injection and data was collected twice weekly. Each study was ended once mean control tumor volume reached approximately 1–1.5 cm^3^; change in TV (ΔTV = TV_final_ − TV_initial_) of each group was compared with the control using the formula %T/C = %(ΔTV _(T)_/ΔTV _(C)_). Statistical analysis was performed using a two-tailed student t-test with Welch’s correction.

### Zebrafish model

Studies in zebrafish were reviewed and approved by the Georgetown University Animal Care and Use Committee.

#### Zebrafish tumor metastasis (ZTM) assay

ACC11 PDX tissue was labeled with viable dye as above and was implanted in 2 dpf zebrafish, as above. Implanted embryos were arrayed in 96-well plates and treated with 1% DMSO or 0.3 μM of Regorafenib. After 5 days, the fish were the number of cells that had migrated into the tail was counted. If ≥ 4 cells migrated, it was scored as high. If 0–3 cells migrated it was scored as low. The percentage of fish that had migrated cells in each group was calculated and plotted in Excel.

#### Extravasation assay

The maximum tolerated dose (MTD) dose for zebrafish was first determined by treating with a range of Regorafenib concentrations or 1% DMSO (control group) in fish water for 7 days. Fish were arrayed in 96 well plates and scored for death and edema daily (Supplementary Fig. 1). A MTD for Regorafenib was determined where there was no death of fish and negligent edema due to drug exposure. Cells (ACC11 and ACC6) were pre-treated with 50 μM of Regorafenib or with 1% DMSO for 45 minutes during the labeling step, washed vigorously and injected in the precardiac sinus of 2dpf zebrfish embryos^60^. All fish were treated with 0.3 μM of Regorafenib in the fish water and observed for extravasating cells daily. Once significant numbers of cells began to extravasate, the number of extravasated cells in each tail was counted. At least 50 fish were injected for each group. The number of extravasated cells in each group was plotted as box plot using Statview 5.01 (SAS Institute, Cary, NC, USA). Paired student T test was performed on the data and *p* value < 0.05 was considered statistically significant.

## Electronic supplementary material

Supplementary Figures
