# Supplementary material for: A multiplex preclinical model for adenoid cystic carcinoma of the salivary gland identifies regorafenib as a potential therapeutic drug
Source: Sci Rep. 2017 Sep 12;7:11410. doi: 10.1038/s41598-017-11764-2 (PMC5595986; doi:10.1038/s41598-017-11764-2)
Supplement: Supplementary file 1 — Supplementary Figures [file 41598_2017_11764_MOESM1_ESM.pdf]

A multiplex preclinical model for adenoid cystic carcinoma of the salivary gland identifies regorafenib as a potential therapeutic drug

Chen Chen[1#], Sujata Choudhury[1#], Darawalee Wangsa[2#], Chamille J. Lescott[1], Devan J. Wilkins[1], Praathibha Sripadhan[1], Xuefeng Liu[1], Danny Wangsa[2], Thomas Ried[2], Christopher Moskaluk[3], Michael J. Wick[4], Eric Glasgow[5], Richard Schlegel[1] and Seema Agarwal[1*]

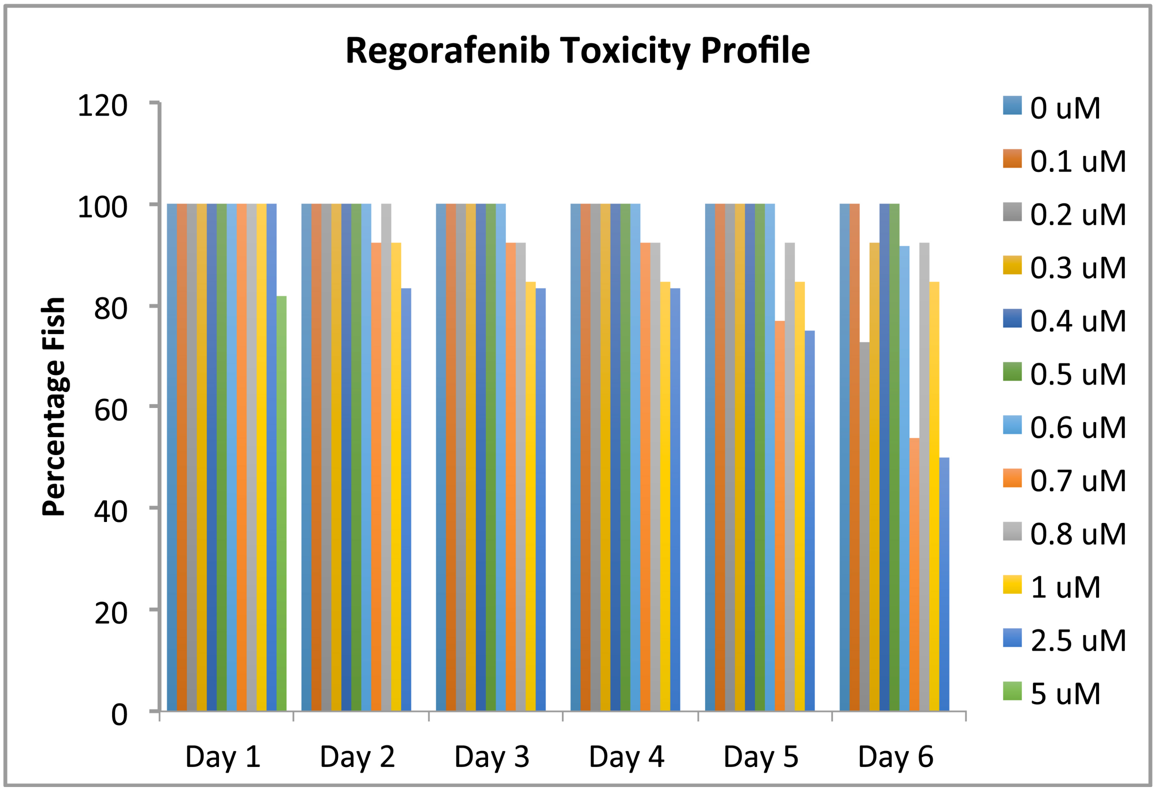

Supplementary Figure 1. Determination of MTD for regorafenib in zebrafish. Fish were continuously exposed to various different concentrations of regorafenib and arrayed in 96-well plate. Fish were scored each day for death and any sign of edema. Percentage of fish alive in any given drug concentration was plotted in excel for each day. 50 fish were used for each drug concentrations.

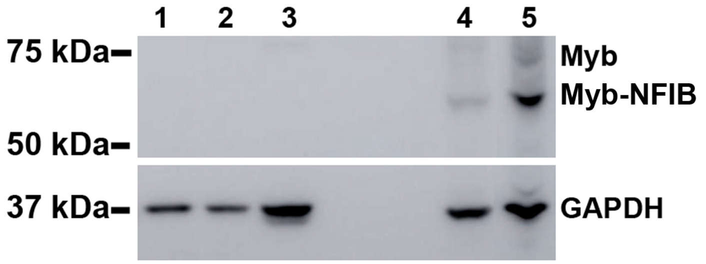

Supplementary Figure 2. Un-cropped image of gel blot for Figure 3C. Lane 4 is deleted in the main figure and the lane 5 in this figure has become lane 4 in the main figure.
